# Assessing the impact of previous experience on lie effects through a transfer paradigm

**DOI:** 10.1038/s41598-021-88387-1

**Published:** 2021-04-26

**Authors:** Claudia Mazzuca, Mariagrazia Benassi, Roberto Nicoletti, Giuseppe Sartori, Luisa Lugli

**Affiliations:** 1 Department of Psychology, University of York, Heslington, York, YO10 5DD UK; 2 Department of Psychology, University of Bologna, Bologna, Italy; 3 Department of Philosophy and Communication, University of Bologna, Via A. Gardino, 23, 40122 Bologna, Italy; 4 Department of General Psychology, University of Padua, Padua, Italy

**Keywords:** Psychology, Human behaviour

## Abstract

Influential lines of research propose dual processes-based explanations to account for both the cognitive cost implied in lying and for that entailed in the resolution of the conflict posited by Simon tasks. The emergence and consistency of the Simon effect has been proved to be modulated by both practice effects and transfer effects. Although several studies provided evidence that the lying cognitive demand may vary as a function of practice, whether and how transfer effects could also play a role remains an open question. We addressed this question with one experiment in which participants completed a Differentiation of Deception Paradigm twice (baseline and test sessions). Crucially, between the baseline and the test sessions, participants performed a training session consisting in a spatial compatibility task with incompatible (condition 1) or compatible (condition 2) mapping, a non-spatial task (condition 3) and a no task one (condition 4). Results speak in favour of a modulation of individual performances by means of an immediate prior experience, and specifically with an incompatible spatial training.

## Introduction

In everyday life, the capacity to flexibly manage our behavior is fundamental. Specifically, the ability to identify situations in which we can automatically respond to stimuli, and those in which instead is necessary to inhibit this automatic response and reprogram an alternative answer is crucial. In fact, only a system with a fully evolved executive control can discern which type of response is most suitable according to the goals to be achieved. The human capability of controlling and reprogramming automatic responses has been examined in cognitive psychology using different tasks. Amongst those, results coming from truth telling/lying tasks and the Simon task contributed to shed light on some of the cognitive mechanisms underlying processes allowing us to flexibly adapt our responses.

Research investigating deception approached it from various perspectives, among which its relation with physiological and cognitive stress. A longstanding tradition conceives physiological stress as a sign of deception and developed numerous techniques over the years to measure it. For instance, the assumption underlying lie detection tests is that lying leads to stress, while telling the truth involves a minor amount of stress, if not at all (e.g., Refs.^1–3^). In this context, facial expressions and tone of voice^4,5^ or increasing blood perfusion resulting in localized elevated temperature (Refs.^6,7^; see also Ref.^8^) are considered to be proxy indicators of deception. More specifically, elevated levels of stress were found to be associated with elevated periorbital perfusion and temperatures^9^. However, not always stress can be associated with deception. In fact, lying can also be strategic and even produce delight, while in some circumstances telling the truth could be stress-inducing (e.g., “Othello error”, see Ref.^2^).

Among the differing approaches employed to study deception, the cognitive perspective on lying has recently gained increasing attention. Within this framework, a consistent amount of studies suggested that lying requires a greater cognitive effort than truth telling, thus leading to longer reaction times in the answers (for a review see Ref.^10^). The increased cognitive load associated with lying has been reported using a variety of measures besides RTs. For example Monaro et al.^11^ demonstrated, by means of mouse trajectories, that unexpected questions increase cognitive load especially for liars (for similar results with keystroke analysis see Ref.^12^). This was reflected in a more diverse pattern of trajectories and in a higher number of errors for liars compared to truth-tellers. Furthermore, brain imaging studies showed that deception involves the activation of executive prefrontal brain regions that are linked to cognitive control and executive functions, while truth telling is not associated with any areas of increased association (Ref.^13^; for reviews see Refs.^14,15^). However, while several studies support a cognitive taxation in lying with respect to telling the truth, other studies show that this pattern can be attenuated or even reversed when taking into account different possible moderators such as—among others—incentives or consequences, showing how composite is the relation between cognitive effort and deception (for a more nuanced, communication-oriented view, see Ref.^16^). Among the many behavioral paradigms used for the study of cognitive processes related to lying, tests measuring response times (RTs) and those allowing for a within subjects comparison between truth telling and lying (e.g., Concealed Information Test, the autobiographical Implicit Association Test, Sheffield Lie Test and the Differentiation of Deception paradigm) have proved to be valid control tools for monitoring the time required to perform specific cognitive operations related to the production of a lie (for a meta-analysis see Ref.^2^).

In order to explain the enhanced cognitive cost of lying, some proposals suggested that lying requires the inhibition of a prepotent truthful response (e.g., Refs.^15,17–19^). According to these perspectives, truthful responses may be considered as a relative baseline, while to respond deceptively the cognitive control needs to resolve a response conflict emerging from the activation of the automatic dominant truth response. So, to account for the increase of RTs found in deceptive responses it has been proposed a dual-process account^20^, in which two task-sets running in parallel are involved. One task-set—based on familiarity—generates an automatic and fast response, while the other task-set—based on recollection—elicits a slower, more controlled response. When participants are required to tell the truth, the two task-sets match and a fast response is produced. On the other hand, when participants are required to lie, a conflict needs to be resolved so that the automatic response has to be inhibited and aborted in favor of a more controlled one—thus requiring a greater cognitive effort (for a truth’s functional role in lying and the proposal of a two-steps process see Ref.^21^).

The capacity to suppress a reflexive response and to emit a more controlled one has been also extensively addressed in studies focusing on spatial attention, such as those employing the Simon task. In a standard Simon task, participants are instructed to press a left or right key in response to the stimulus color (relevant dimension) ignoring its lateralized position (irrelevant dimension). Converging evidence showed that performances, both in terms of speed and accuracy, are more efficient when the position of the responses and stimuli is ipsilateral (corresponding trials) than when it is contralateral (non-corresponding trials), despite the fact that the position of the stimulus is irrelevant to correctly complete the task. The advantage of the corresponding over the non-corresponding trials is known as Simon effect (Refs.^22–25^; for reviews, see Refs.^26,27^).

Similarly to proposals explaining the cognitive cost implied in lying, the dual route process is also widely used to account for the Simon effect (e.g., Ref.^28^). A large amount of evidence in fact supports the view that the Simon effect is the result of the interaction between two parallel and independent processing routes. When the stimulus appears, the response is automatically activated in the direct route by the stimulus position thanks to pre-existing, long-lasting stimulus–response (S–R) associations, which are independent from the current instruction. In contrast, the indirect route activates the required response based on the task-defined associations connecting the relevant dimension of the stimulus (e.g., the blue color) to the specific response. While in corresponding trials the automatic and the required response correspond and no conflict occurs, the opposite holds for non-corresponding trials where the automatic response needs to be inhibited and aborted, causing a slowing of RTs and a decrease in accuracy.

Several lines of evidence suggest that the cognitive cost entailed both in responding to non-corresponding trials and in lying can be modulated, respectively resulting in reduced Simon and lie-effects (i.e., the difference in RTs between truth-trials and lie-trials, see Ref.^29^).

For instance, the emergence and consistency of the Simon effect has been proved to be modulated by both practice (e.g., Refs.^30,31^) and transfer effects (e.g., Refs.^32,33^). Practice effects refer to the decreasing of the Simon effect as a function of the number of trials practiced by participants. For example, Proctor and Lu (Ref.^30^, Experiment 1) asked participants to perform a series of 1800 Simon trials divided into three sessions. Results showed that the Simon effect decreased from the first session (22 ms) but persisted at a reduced magnitude until the last sessions (14 ms). The authors explained the reduction of the Simon effect suggesting that participants, by practicing with the same type of trials, might have learnt to ignore or to suppress the irrelevant information of stimulus location. Transfer effects are instead intended as the modulation of the Simon effect by means of a prior spatial training task. For instance, when participants perform a spatial compatibility task with a compatible Stimulus–Response (S–R) mapping (i.e., pressing the right key if the stimulus appears on the right and the left key if the stimulus appears on the left) in the training session, and a standard Simon task in the test session, the Simon effect remains unaffected (e.g., Ref.^34^, Experiment 2). In contrast, when a spatial compatibility task with an incompatible S–R mapping is employed in the training session (i.e., pressing the right key if the stimulus appears on the left and the left key if the stimulus appears on the right), in the test session the Simon effect is reduced, eliminated, or even reversed (e.g., Ref.^30^, Experiment 2 and 3^32–36^). This has been explained suggesting that new, task-related, non-corresponding S–R associations are acquired and trained in the training session and remain active enough to influence the Simon effect in the subsequent test session. This new association would compete with the spatially corresponding response, and the Simon effect would be affected. These results are intriguing, in that they show that the modulation of individual performances in a standard Simon task can be affected not only by the goals of the task, but also by immediate prior experience with a different yet related task—as in the case of incompatible spatial training—that can impact the stability of the Simon effect.

Interestingly, recent evidence suggests that also the cognitive demand associated with lying can be reduced by means of practice effects—although the notion of practice effect slightly differs from the one usually accounting for the Simon effect. In fact, in the deception literature the practice effect is usually assessed by manipulating the proportion between lie and truth trials, or giving participants some kind of incentive through specific instructions. For example, it has been demonstrated that (1) practice generally decreases the response times of deceptive responses presented in blocks in a working memory task^37^; (2) when participants are allowed to prepare and practice their lies before a lie detection test, their deceptive responses are associated with reduced response times^38^; (3) manipulating the proportion of truthful or deceptive responses has a selective positive effect on lying, so that “frequent lying made lying easier whereas frequent truth telling made lying more difficult” (Ref.^39^, p. 909). Along these lines, Hu et al.^40^ employed a two-sessions Differentiation of Deception (DoD) paradigm in which participants had to respond truthfully (one block) or deceitfully (another block). Crucially, the authors manipulated the tasks performed between the two DoD sessions instructing three groups differently: (1) a ‘control’ group performed an irrelevant vision illusory task; (2) an ‘instruction’group was debriefed about their performance in the first session and then encouraged to try their best to speed up their RTs in the second session; and (3) a ‘training’ group followed the same procedure of the instruction group, so they were required to speed their performance, but they were also given 360 additional deceptive trials to practice their deceptive responses. Results showed that for both the ‘instruction’ and the ‘training’ group, in which participants intentionally acted with the conscious goal of speeding up their responses, the cognitive demand associated with deception decreased. Interestingly though, only for the ‘training’ group the difference between deceptive and truthful responses disappeared. So, while in the Simon task the standard practice effect emerges by increasing overall the amount of trials, in lying tasks this is variously measured. Furthermore, findings regarding the effect of practice on the lie-effect measured by means of RTs are disputed (see Ref.^2^).

To sum up, the cognitive demand connected with lying may vary as a function of practice, but whether and how transfer effects could also play a role—as in the case of Simon effect—remains an open question. In fact, given that dual-processes based explanations have been proposed to account for both the enhanced cognitive cost of deception, and for that accounting for the Simon effect, one can ask whether there may be similar underlying cognitive processes. If that is the case, it is possible that the lie-effect could benefit from the manipulations that have been found to affect the Simon effect. Here, we directly investigated whether and how individual performances on a truth telling/lying task could depend not only on the goals of the current task, but also on immediate previous specific experiences, as suggested by studies concerned with the Simon effect. More specifically, we tested if the cognitive cost of deception—as measured by RTs—could be reduced by training a specific spatial learning in an immediate prior experience.

With this purpose, we asked participants to complete a Differentiation of Deception Paradigm, a relatively superficial task, consisting in a Baseline and a Test session that were identical. Crucially, though, participants were assigned to four different conditions matching four different tasks in the session between the Baseline and the Test, i.e., a Training session. In the first two conditions, participants were required to perform a Training consisting in a spatial compatibility task with incompatible (condition 1) or compatible (condition 2) mapping (as in the standard studies on the transfer effect with the Simon task). Conditions 3 and 4 were designed as control conditions. In condition 3, a non-spatial training session was run and in condition 4, there was no training session.

We expected to find a different modulation of the lie-effect in the first condition compared to the other three conditions due to the transfer effect emerging as a function of the specificity of training. Specifically, we hypothesized to find a decrease of the lie-effect when the Training task required an incompatible mapping (condition 1). In fact, if it is true that the liar would need to first inhibit and suppress the truthful response, and then produce the lie, then a previous experience in which an incompatible mapping is trained should reduce the cost required for this two-steps process. We did not specifically predict the lie-effect to be affected by practice for two main reasons. First, as already discussed, the evidence showing an influence of practice effects on lie-effect is conflicting; second, our manipulation is not comparable to the ones generally employed in detection studies to test the effect of practice.

## Results

In order to guarantee the statistical validity of subsequent analyses we reduced the skewness of the dependent variable by transforming RTs into natural logarithms.

To explore the relation between our variables of interest (Session: baseline vs test; Training: incompatible spatial vs compatible spatial vs non-spatial vs no task; and Instruction: lie vs truth) we fitted two linear mixed models^41^ with the log-transformed RTs as dependent measure, and participants and items as random factors. The two models vary for the complexity of fixed effects. We first present the results of the model directly testing our hypothesis, and then turn to the discussion of the explorative saturated model.

### Model 1

We fitted a linear mixed model with the log-transformed RTs as dependent variable, Session (baseline vs test), Training (incompatible, compatible, non-spatial, no task), and Instruction (lie vs truth) as fixed effects, as well as the interaction between the three factors, and participants and items as random effects. Akaike information criterion (AIC)^42^ indicated a fit of 5777, whereas Bayesian information criterion (BIC)^43^ indicated a fit of 5889.4. We found a significant main effect of Session *F*(1, 8241.2) = 109.32, *p* < 0.001, showing reduced response times from the baseline session (EMM = 7.74; SE = 0.112; LCI = 7.28; UCI = 8.20) to the test session (EMM = 7.53; SE = 0.112; LCI = 7.08; UCI = 7.99). We also confirmed the general advantage of truthful responses (EMM = 7.53; SE = 0.112; LCI = 7.08; UCI = 7.99) over deceitful responses (EMM = 7.74; SE = 0.112; LCI = 7.28; UCI = 8.20), *F*(1, 8241.9) = 189.11, *p* < 0.001. In addition, we found a significant main effect of Training, *F*(3, 84.3) = 3.37, *p* = 0.022, showing that overall participants in condition 1 were slightly faster than participants in the other conditions (condition 1 EMM = 7.57; SE = 0.117; LCI = 7.15; UCI = 7.99; condition 2: EMM = 7.60; SE = 0.117; LCI = 7.19; UCI = 8.02; condition 3: EMM = 7.66; SE = 0.117; LCI = 7.25; UCI = 0.08; condition 4: EMM = 7.70; SE = 0.117; LCI = 7.28; UCI = 8.12).

We also found a significant three-way interaction between Session, Instruction, and Training*, F*(7, 8241.7) = 2.31, *p* = 0.023.

### Model 2

We fitted a linear mixed model with log-transformed RTs as dependent measure, Session (baseline vs test), Training (incompatible, compatible, non-spatial, no task), and Instruction (lie vs truth) as fixed effects, and participants and items as random factors. In this Model 2, we included in the model all two-ways interactions (Session × Training; Session × Instruction; Instruction × Training) as well as the interaction between the three factors (Session × Training × Instruction). AIC indicated a fit of 5779.7, whereas BIC indicated a fit of 5913.2. We found a significant main effect of Session *F*(1, 8241.5) = 770.037, *p* < 0.001, showing reduced RTs in the test session (EMM = 7.53; SE = 0.112; LCI = 7.08; UCI = 7.99) compared to the baseline session (EMM = 7.74; SE = 0.112; LCI = 7.28; UCI = 8.20). We also found a significant main effect of Training *F*(3, 84.4) = 3.28, *p* = 0.024, showing that overall participants in condition 1 were slightly faster than all the other conditions (condition 1 EMM = 7.57; SE = 0.117; LCI = 7.15; UCI = 7.99; condition 2: EMM = 7.60; SE = 0.117; LCI = 7.19; UCI = 8.02; condition 3: EMM = 7.66; SE = 0.117; LCI = 7.25; UCI = 0.08; condition 4: EMM = 7.70; SE = 0.117; LCI = 7.28; UCI = 8.12). Finally, we found a significant main effect of Instruction, *F*(1, 8241.9) = 189.131, *p* < 0.001, showing a general advantage of truthful responses (EMM = 7.53; SE = 0.112; LCI = 7.08; UCI = 7.99) over deceitful responses (EMM = 7.74; SE = 0.112; LCI = 7.28; UCI = 8.20). We found no other significant main effect or interaction (all *F*_s_ < 2.126; all *p*_s_ > 0.094).

Likelihood ratio tests comparing Model 1 with Model 2 showed no significant difference between the two models, ${\rm X}$^2^(3) = 3.309, *p* = 0.346. Given that both the AIC and the BIC criteria indicated a better fit for Model 1 with respect to Model 2, and that Model 1 estimated less parameters than Model 2, we can confidently interpret Model 1 as better explaining our data.

To further explore the three-way interaction we found in Model 1, and to assess whether the incompatible condition (condition 1) specifically affected our dependent variable, we performed Helmert’s contrasts. The first contrasted condition 1 (incompatible spatial training) against the average of the next three conditions (compatible spatial training, non-spatial training, and no-task); the second contrast compared condition 2 (compatible spatial training) against the average of following two conditions (non-spatial training, no-task); the third contrast compared condition 3 (non-spatial task) against condition 4 (no-task).

The first contrast showed that condition 1 significantly differed from the subsequent conditions, *t*(22.86) = − 2.698, *p* = 0.008. The other two contrasts instead revealed no significant differences. Condition 2 (compatible spatial training) did not differ from the average of the next two conditions, *t*(23) = − 1.669, *p* = 0.098, nor did condition 3 (non-spatial training) from condition 4 (no-task), t(22.87) = − 0.231, *p* = 0.818.

To better understand the direction of this effect, in line with previous studies (see e.g., Ref.^29^), we computed lie-effects in each condition by subtracting the RTs of truth-trials from the RTs of lie-trials. Here, we use RTs to allow for a clearer interpretation of results. We separately computed paired sample t tests for each condition, comparing the lie effect of the Baseline session to the one of the Test session. An open-source tool was used to compute Cohen’s *d*_*z*_ effect size for the *t* tests (https://www.uccs.edu/lbecker/).

Errors (condition 1: 10.8% and 8%, condition 2: 13% and 7.7%, condition 3: 11.7% and 8.7%, condition 4: 10.9% and 7.2% of the total trials for the baseline and the test sessions, respectively) were excluded from the analysis on RTs.

**Figure 1:**
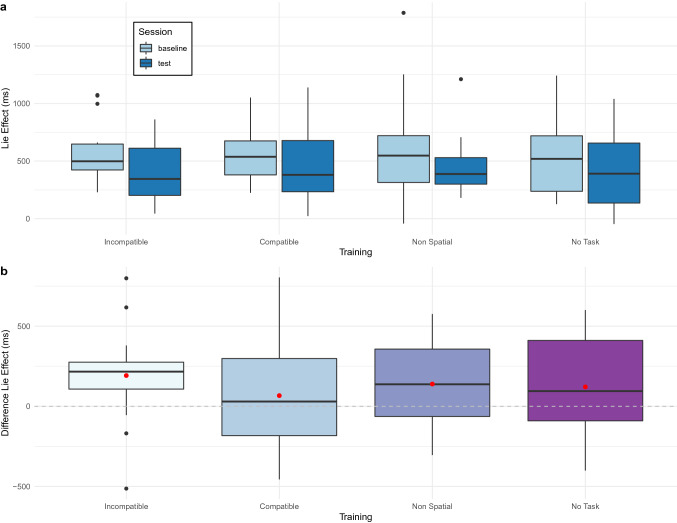
(**a**) Boxplots of lie-effect in the baseline and the test sessions for each condition. Black bars represent medians, while black dots represent extreme values. (**b**) Boxplots of mean differences of lie-effect between the baseline and the test session for each condition. A grey dashed line indicates no difference, black bars represent medians, red dots represent means, and black dots represent extreme values.

For condition 1 (training with an incompatible spatial task), we found that the lie effect differed between the two sessions, *t*(15) = 2.551, *p* = 0.022, *d*_*z*_ = 0.77. It significantly decreased from the baseline session (584 ms) to the test session (392 ms). In contrast, for the other three conditions the lie effect did not differ between the two sessions, as showed by the paired-sample t tests: *t*_*s*_(15) = 0.763, 1.889 and 1.488, *p*_*s*_ = 0.457, 0.078 and 0.158, *d*_*z*_s = 0.23, 0.38 and 0.37, for the training with a compatible spatial task (543 ms and 476 ms for the baseline and test session, respectively), non-spatial training (591 ms and 452 ms for the baseline and test session, respectively) and no-training conditions (539 ms and 418 ms for the baseline and test session, respectively), see Fig. 1.

## Discussion

Among many other cognitive processes, the human ability to lie has interested the scientific community for a long time. This enduring interest led to the development of diverse methodologies aimed at detecting deception, among which measuring response times of deceitful responses. Cognitive theories on lying and truth telling posit that lying takes longer than truth telling. Despite most research done to date found a variation of the lie effect using different manipulations (e.g., practiced deception, relevant information, and motivated liars^19,44,45^), no studies reported a reversed RT lie-effect, suggesting that the cognitive cost implied in lying is a robust and stable phenomenon^2^. For example, Van Bockstaele et al.^29^ investigated whether practice in lying or telling the truth influences the cognitive cost of lying. Their results show that, in the training phase, lying became more difficult for participants in the frequent-truth group than for participants in the frequent-lie group. Although these results are in line with Verschuere et al.^39^, they are in conflict with, for example, Johnson et al.^37^, and Vendemia et al.^46^, indicating that a heated debate on the resistance against practice of the cognitive cost of lying is currently ongoing.

Interestingly for the purpose of this study, not only the literature is inconclusive on the stability and robustness of the lying cognitive cost, but most of the studies examined the cost of lying by asking participants to practice with the same type of trials (for instance incrementing the number of lie trials; i.e., practice effect).

In our study, following Van Bockstaele et al.^29^’s procedure, participants were faced with a baseline, a training and a test session. Differently, though, in the training session the task no longer involved responding truthfully or deceptively to the same set of trials: participants responded to spatial (condition 1 and 2) or perceptive (condition 3) dimensions of the stimuli. We hypothesized that performing an incompatible spatial training between the two sessions (e.g., condition 1) would have trained the inhibition of automatic responses, so our aim was to assess whether this training effect would be carried over to the last session—thus modulating the lie-effect.

Our results spoke in favor of this expectation. We found that condition 1 significantly differed from the other three conditions, suggesting that the specificity of the training performed differentially affected RTs. More specifically, only in the group performing a spatial incompatibility task (condition 1) we found a significantly reduced lie-effect from the baseline to the test session. In contrast, although we observed a numerical decrease of the lie-effect in the other three conditions, the difference between the lie-effect in the baseline and in the test session did not reach significance, suggesting that the lie-effect was not modulated by the prior training. Importantly, although the task employed (DoD) might be regarded as shallower than tasks requiring e.g., message/linguistic production (see e.g., Ref.^16^), two general conclusions concerning the cognitive cost associated with lying can be drawn from our results.

First, it is crucial to acknowledge the difference between practice and transfer effects. Previous studies on the lie-effect typically implementing a practice paradigm (e.g., manipulating the proportion of lie trials) revealed no conclusive results on the possible modulation of the cognitive cost of lying. Here, we implemented a paradigm informed by the literature on Simon effect, in which we did not manipulate the proportion of lie and truth trials, but we simply presented participants with two identical DoD sessions, composed of an equal number of lie and truth trials. Importantly, though, we manipulated the type of task occurring between the two sessions. Our results provide preliminary evidence that the mere repeated exposure to the same proportion of lie and truth trials is not sufficient for reducing the lie-effect. In fact, even though we found a numerical reduction of the lie-effect in all the four conditions, only in the condition in which the training was meant to exercise an incompatible mapping (condition 1), the lie-effect significantly decreased. It is perhaps worth noting that, while the literature on the relation between practice and lying is growing, the possible interplay between deception and transfer effects is still unexplored. In this context, our study provides preliminary results that can inform future research deepening these aspects.

Second, the present results add some insights on the mechanisms underlying the enhanced cognitive cost of deception. More specifically, our results seem to support the assumption that an active response inhibition is required to overcome the prepotent truthful response (e.g., Refs.^15,17–19^). In fact, only when the training session instructed participants to learn to inhibit an automatic response and to come out with an incompatible one (condition 1), the lie-effect was affected. Being trained to respond incompatibly, participants carried over the newly acquired non-corresponding S–R associations related to the spatial task, that remained active enough to genuinely alter the lie-effect in the subsequent test session.

The insights gained from this study may be of assistance to the literature addressing whether and how the lie-effect could be modulated. Indeed, if the lie-effect can be modulated for example by an incompatible spatial training, then telling the truth and lying may not depend only on the task that is currently being performed, but also on the immediate prior experience, even when the activity required is completely different.

In a recent study, Osman et al.^47^ followed the same logic but from the opposite point of view. The authors investigated if the experience of responding deceptively could influence a non-deceptive task that required similar incongruence processes, such as the Stroop task. In their Experiment 2, Osman and colleagues showed that a task-specific training, exercising “incongruent” responses such as lying, affected participants' performances in a subsequent different task such as the Stroop task. Specifically, after practicing in responding deceptively, participants showed a facilitation in the incongruent Stroop trials, suggesting a potential link between the two underlying mechanisms of suppression and activation of automatic responses.

Similar approaches highlighting potential similarities between the process of lying and more well documented effects could be taken, to fully clarify and deepen the general underlying mechanisms involved in deception. The findings from our study call for further attention on the transfer effects of specific paradigms on subsequent tasks that could share the same underlying processes, in order to better comprehend why lying seems to be cognitively more demanding than truth telling.

## Methods

### Data availability

The datasets generated and analyzed during the current study are available in the Open Science Framework repository, https://osf.io/rv7sk/.

### Participants

Sixty-four students from the University of Bologna (45 females, 8 left-handed, *M*_age_ = 21.5, *SD*_age_ = 3.19) voluntarily took part in the study. All reported normal or corrected-to-normal vision and were naïve as to the purpose of the experiment. Once recruited, participants were randomly assigned in equal proportions to one of four experimental conditions (16 participants for each condition).

### Ethics statement

The experiment included in this study was performed in accordance with the ethical standards laid down in the Declaration of Helsinki and fulfilled the ethical standard procedure recommended by the Italian Association of Psychology (AIP). The present study is part of a wide project entitled “Compatibility between different types of stimuli and related responses”, approved by The Ethic Committee of the University of Bologna.

All participants gave their written informed consent to participate in the study and they were debriefed about the aim of the study at the end of the experiment.

### Apparatus and stimuli

The experiment took place in a dimly lit and noiseless room. Stimulus presentation, response recording, and data collection were controlled with the E-Prime 2.0 software (http://www.pstnet.com). Participants were seated facing a 17-in. cathode-ray tube screen at a viewing distance of 60 cm.

For the Baseline and Test sessions, a Differentiation of Deception Paradigm (DoD, see Ref.^48^) was used. Seventy-two sentences, half about general knowledge and half related to the current context were used. Half of the sentences about general knowledge were correct (e.g., “The pound is the English currency”) and half of them were wrong (e.g., “Bees produce ricotta”), and the same was true for the sentences related to the current context (e.g. “I’m at the University” and “I’m in Australia” for correct and wrong sentences, respectively). At the beginning of the block, 12 different sentences served as practice (see the Supplementary Information for the complete list of sentences used). Sentences were written in white ink and presented at the center of a black screen. Words were written in a 21-point Courier New font.

For the Training session consisting in the spatial compatibility tasks (i.e., condition 1 and 2), stimuli were white squares (3.8° × 3.8°) presented to the left or to the right of a centered fixation cross (0.95° × 0.95°) on a black screen. For the Training session consisting in the non-spatial task (i.e., condition 3) the stimuli were the same used in the previous conditions but colored in blue or red and presented in the center of the screen.

### Procedure

Participants were asked to complete a task divided into three sessions. In the first (Baseline) and third (Test) sessions participants were instructed for half of the trials to lie and for the other half to tell the truth about the sentences presented, depending on a cue (the word “lie” or “truth” presented randomly just before the sentences). It is important to underline that lies can be of different types and variable cognitive loads. Since this study aims to investigate an elementary form of lie, the task required a simple reverse response.

**Figure 2:**
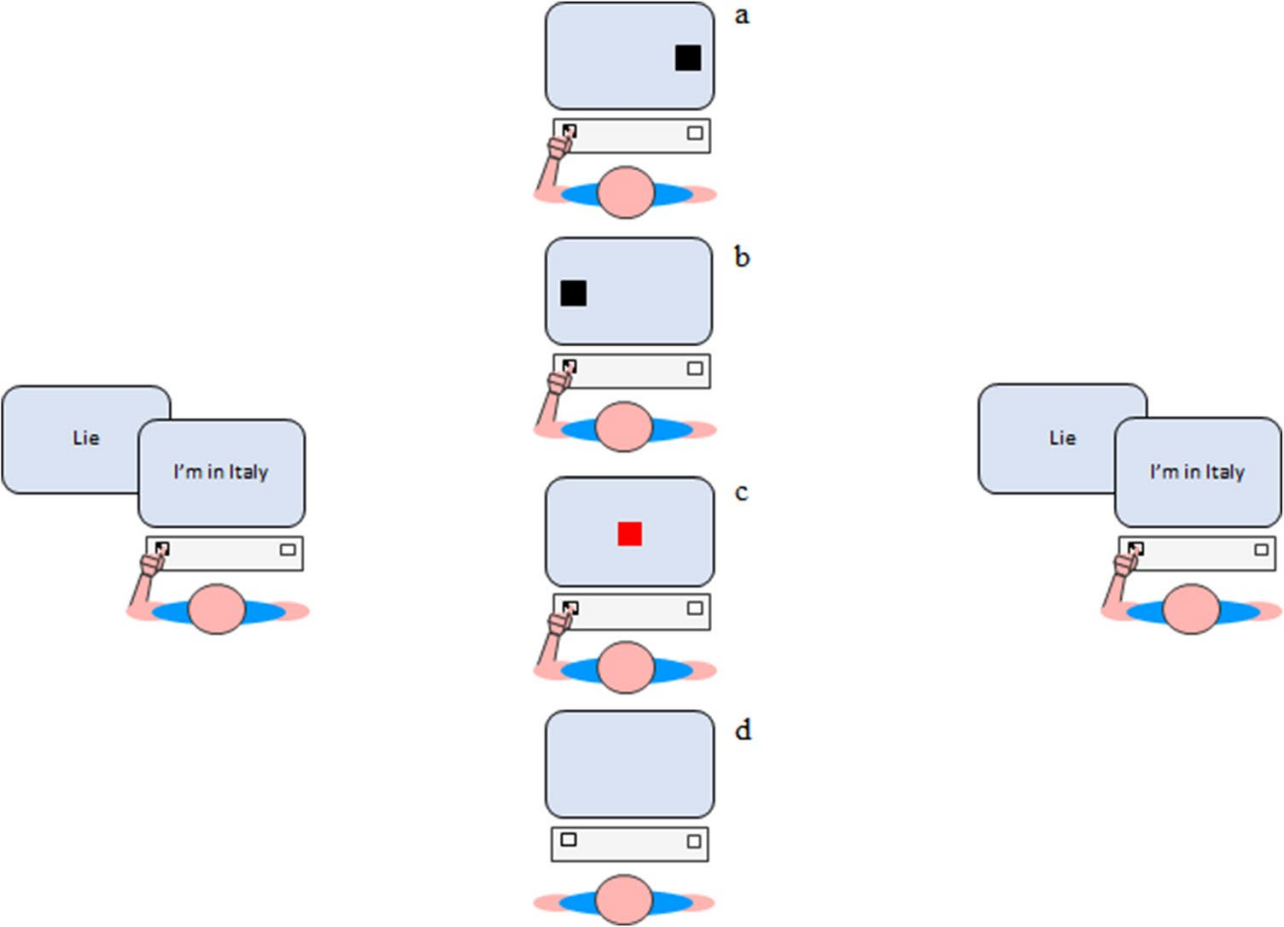
Temporal sequence of a representative trial in the Baseline (leftmost panel) and in the Test sessions (rightmost panel), and during the Training session [middle panel (**a**): incompatible spatial task, condition 1; middle panel (**b**): compatible spatial task, condition 2; middle panel (**c**): non-spatial task, condition 3; middle panel (**d**): no-Training task, condition 4]. Note that stimuli are not drawn to scale.

Participants responded by pressing two keys on a QWERTY keyboard: the right button (the “–” key) for the “true” responses and the left button (the “Z” key) for the “lie” responses (see Fig. 2, left and right panel for Baseline and Test sessions, respectively).

In the Baseline and in the Test sessions, the trial started with a fixation presented at the center of the screen for 1000 ms. Subsequently, the instruction (the word “lie” or “truth”) was presented for 1000 ms and then the stimulus appeared and remained on the screen until a response was provided. The two sessions consisted of 72 experimental trials preceded by 12 trials of practice.

The second session (Training) varied depending on the four conditions. In the Training session of condition 1, participants were asked to carry out a spatial compatibility task with an incompatible Stimulus–Response (S–R) mapping [for a deep analysis of the motor preparation see^49^], that is to respond as quickly and accurately as possible to the left stimulus by pressing the right response key and to the right stimulus by pressing the left response key (left and right buttons were the same used in the first and the third session), see Fig. 2, middle panel a. In the Training session of condition 2, participants were required to perform a spatial compatibility task with a compatible S–R mapping. They were instructed to respond as quickly and accurately as possible to the left stimulus by pressing the left response key and to the right stimulus by pressing the right response key (see Fig. 2, middle panel b). In the Training session of condition 3, half of participants were instructed to respond as quickly and accurately as possible to the blue stimulus by pressing the right response key and to the red stimulus by pressing the left response key (see Fig. 2, middle panel c), while the other half experienced the opposite mapping rule. In condition 4, the Training session was not present. Participants waited 5 min without doing any kind of task and then started the Test session (see Fig. 2, middle panel d).

In the Training session of the first three conditions, the trial started with a fixation presented at the center of the screen for 1000 ms. Subsequently, the stimulus appeared and remained on the screen until a response was provided. The Training session consisted of 150 experimental trials that were divided into three blocks of 50 trials each and preceded by 10 trials of practice.

## Supplementary Information

Supplementary Information.
