# Supplementary material for: Assessing the impact of previous experience on lie effects through a transfer paradigm
Source: Sci Rep. 2021 Apr 26;11:8961. doi: 10.1038/s41598-021-88387-1 (PMC8076267; doi:10.1038/s41598-021-88387-1)
Supplement: Supplementary file 1 — Supplementary Information. [file 41598_2021_88387_MOESM1_ESM.pdf]

Supplementary Information

Claudia Mazzuca[1], Mariagrazia Benassi[2], Roberto Nicoletti[3], Giuseppe Sartori[4], Luisa Lugli[3*]

[1]Department of Psychology, University of York, York, United Kingdom

[2]Department of Psychology, University of Bologna, Bologna, Italy

[3]Department of Philosophy and Communication, University of Bologna, Bologna, Italy

[4]Department of General Psychology, University of Padua, Padua, Italy

Complete list of sentences used as stimuli

12 Practice trials			
**Type of Sentence**		**Original version (Italian)**	**English Translation**
**General Knowledge**	**Correct**	A calcio si gioca con il pallone	Soccer is played with the ball
		Il burro si tiene in frigorifero	The butter is kept in the refrigerator
		La televisione si guarda con gli occhi	The television is watched with the eyes
	**Wrong**	Ho meno di 15 anni	I am under 15 years old
		Sono alto più di due metri	I am taller than 2 meters
		Sto aspettando l'autobus	I'm waiting for the bus
**Current context**	**Correct**	Ho due occhi e un naso	I have two eyes and a nose
		Ho meno di 50 anni	I am under 50 years old
		Sono un essere umano	I am a human being
	**Wrong**	Ho meno di 15 anni	I am under 15 years old
		Sono alto più di due metri	I am more than two meters tall
		Sto aspettando l'autobus	I'm waiting for the bus

72 Experimental trials			
**Type of Sentence**		**Original version (Italian)**	**English Translation**
**General Knowledge**	**Correct**	Il brodo si serve in un piatto fondo	The broth is served in a soup plate
		Il cane offre la zampa	The dog offers the paw
		Il dattero cresce sulla palma	The date grows on the palm tree
		Il gatto ha quattro zampe	The cat has four legs
		Il naso è vicino agli occhi	The nose is close to the eyes
		In una camicia i gemelli si mettono ai polsini	In a shirt the cufflinks are worn on the cuffs
		L'Italia confina con la Francia	Italy borders with France
		La mucca produce il latte	The cow makes milk
		La Pasqua è un giorno festivo	Easter is a public holiday
		La poesia è scritta in versi	Poetry is written in verses
		La Sardegna è un'isola italiana	Sardinia is an Italian island
		La sterlina è la moneta inglese	Pound is the English currency
		L'automobile ha quattro ruote	The car has four wheels
		L'oca ha le piume	The goose has feathers
		Nella torta ci metti lo zucchero	An ingredient in the cake is sugar
		Otto è un numero pari	Eight is an even number
		Prima della quaterna si può fare terno	Before the quaterna can be made terno
		Uno starnuto fa rumore	A sneeze makes noise
	**Wrong**		
		Attorno al collo ci si mette i guanti	Gloves are worn around the neck
		Due più due fa sette	Two plus two equals seven
		Gli sci hanno le ruote	Skis have wheels
		Il canguro è capace di volare	The kangaroo is capable of flying
		Il maiale è un equino	The pig is an equine
		Il Natale è un giorno feriale	Christmas is a weekday
		Il sughero nell'acqua affonda	In the water the cork sinks
		In inverno ci sono 40 gradi	In winter there are 40 degrees
		In teatro il loggione si trova in platea	In the theater the gallery is located in the stalls
		L'idraulico lavora lontano dall'acqua	The plumber works far from the water
		La brace sta sopra la griglia	The embers are above the grill
		La Sicilia è un'isola australiana	Sicily is an Australian island
		Le api producono ricotta	Bees make ricotta
		Lo stretto di Messina si può attraversare a piedi	The Strait of Messina can be crossed on foot
		Nella crema pasticcera ci metti il sale	An ingredient of the custard is salt

		Nella lavastoviglie metti il bucato	It is necessary to put the laundry in the dishwasher
		Nove è un numero pari	Nine is an even number
		Per andare a sciare vai al mare	To go skiing go to the sea
**Current context**	**Correct**	Ho due dita sulla tastiera	I have two fingers on the keyboard
		Ho due gambe e due braccia	I have two legs and two arms
		Ho più di 10 anni	I am over 10 years old
		Ho ricevuto delle istruzioni	I have received instructions
		Indosso degli abiti	I wear clothes
		Indosso le scarpe	I wear shoes
		Sono a Bologna	I'm in Bologna
		Sono alto più di un metro	I'm taller than one meter
		Sono davanti al computer	I'm in front of the computer
		Sono davanti allo schermo	I'm in front of the screen
		Sono dentro ad una stanza	I'm in a room
		Sono in Emilia Romagna	I'm in Emilia Romagna
		Sono in Italia	I'm in Italy
		Sono in università	I'm at the university
		Sto facendo un esperimento	I'm doing an experiment
		Sto osservando uno schermo	I am looking at a screen
		Sto usando il computer	I am using a computer
		Sto usando la tastiera	I am using the keyboard
	**Wrong**		
		Ho meno di 10 anni	I am under 10 years old
		Indosso i guanti	I wear gloves
		Indosso un casco	I wear a helmet
		Sono davanti alla tv	I'm in front of the TV
		Sono dentro all'ascensore	I'm in the elevator
		Sono disteso a letto	I am lying in bed
		Sono in aereo	I'm on the plane
		Sono in Australia	I'm in Australia
		Sono in ospedale	I'm in the hospital
		Sono in un parco	I'm in a park
		Sono in una stanza d'albergo	I'm in a hotel room
		Sono negli Stati Uniti	I'm in United States
		Sono sotto ad un albero	I'm under a tree
		Sto bevendo una coca cola	I am drinking a coke
		Sto correndo	I'm running
		Sto scalando un monte	I am climbing a mountain
		Sto scrivendo su un foglio	I am writing on a sheet
		Sto telefonando	I am making a phone call

Control Models

Model 3

We fitted a linear mixed model with the log-transformed RTs as dependent measure, participants and items as random factors, Session and Training as fixed effect, along with their interaction (AIC= 6560.2; BIC= 6586.0). We found that the two variables were poorly correlated (VIF= 1.62).
We found a main effect of Session, $F(1, 8241.5)= 675.32$, $p<.001$, and Training, $F(3, 87.1)= 675.32$, $p=.019$, but no significant interaction between the two terms, $F(3, 8241.5)= 1.93$, $p=.121$.

Model 4

We fitted a linear mixed model with the log-transformed RTs as dependent measure, participants and items as random factors, Instruction and Training as fixed effect, along with their interaction (AIC=6508.7; BIC=6586.0). We found that the two variables were poorly correlated (VIF= 1.15).
We found a main effect of Instruction, $F(1, 8242.4)= 730.68$, $p<.001$. We did not find a significant main effect of Training, $F(3, 63.1)= 2.51$, $p=.066$, nor of the interaction of the two terms, $F(3, 8242.4)= 2.25$, $p=.080$.

Model 5

We fitted a linear mixed model with the log-transformed RTs as dependent measure, participants and items as random factors, Instruction and Session as fixed effect, along with their interaction (AIC=5781; BIC=5830.2). We found that the two variables were moderately correlated (VIF= 2.48).
We found a main effect of Instruction, $F(1, 8241.9)= 118.61$, $p<.001$, and Session, $F(1, 8241.5)= 767.25$, $p<.001$, but no significant interaction between the two terms, $F(1, 8241.6)= 1.32$, $p=.25$.
